# Combined Proteomic and Metabolomic Analyses Reveal the Comprehensive Regulation of *Stropharia rugosoannulata* Mycelia Exposed to Cadmium Stress

**DOI:** 10.3390/jof10020134

**Published:** 2024-02-07

**Authors:** Qin Dong, Mingjie Chen, Changxia Yu, Yaru Zhang, Lei Zha, Pattana Kakumyan, Huanling Yang, Yan Zhao

**Affiliations:** 1 Institute of Edible Fungi, Shanghai Academy of Agricultural Sciences, Shanghai 201403, China; maomao88719@163.com (Q.D.); mjchen@saas.sh.cn (M.C.); ycx41529@163.com (C.Y.); lichforce@126.com (Y.Z.); zhalei@saas.sh.cn (L.Z.); yanghuanling@saas.sh.cn (H.Y.); 2 School of Science, Mae Fah Luang University, Chiang Rai 57100, Thailand; pattana.kak@mfu.ac.th

**Keywords:** *Stropharia rugosoannulata*, Cd stress, proteomics, metabolomics, molecular networks

## Abstract

The potential of *Stropharia rugosoannulata* as a microbial remediation material for cadmium (Cd)-contaminated soil lies in its capacity to absorb and accumulate Cd in its mycelia. This study utilized the TMT and LC−MS techniques to conduct integrated proteomic and metabolomic analyses with the aim of investigating the mycelial response mechanisms of *S. rugosoannulata* under low- and high-Cd stresses. The results revealed that mycelia employed a proactive defense mechanism to maintain their physiological functions, leading to reduced sensitivity to low-Cd stress. The ability of mycelia to withstand high levels of Cd stress was influenced primarily by the comprehensive regulation of six metabolic pathways, which led to a harmonious balance between nitrogen and carbohydrate metabolism and to reductions in oxidative stress and growth inhibition caused by Cd. The results provide valuable insights into the molecular mechanisms involved in the response of *S. rugosoannulata* mycelia to Cd stress.

## 1. Introduction

Soil is an important component of the ecological environment, and human survival relies on cultivated land. Due to the rapid industrial development in China, a large amount of pollutants have been introduced into the soil. A soil pollution survey conducted in 2014 revealed that 16.1% of China’s soil was contaminated by heavy metals, and 7% of the soil is contaminated by cadmium (Cd) [1,2]. Industrial activities, sewage irrigation, livestock manure, phosphate fertilizer application, and atmospheric sedimentation can cause a large amount of Cd accumulation in the soil, and acid rain increases the solubility of Cd^2+^, leading to an increase in Cd toxicity and eventually polluting the soil [3,4,5,6]. Cd is a nonessential element and has high toxicity due to its relatively strong mobility, high chemical activity, long half-life, and resistance to degradation. Once Cd is absorbed by plant roots, it can be translocated throughout the whole plant, where it inhibits a series of physiological and biochemical metabolic processes, including photosynthesis, respiration, and nitrogen and carbon metabolism; thus, it compromises the normal growth and development of plants [7,8,9]. In addition, the human body can accumulate Cd from the food chain, which can negatively impact human health, as its half-life in the human body lasts between 10 and 30 years [10,11]. If the Cd concentration in soil exceeds China’s soil environmental quality standard for Cd pollution risk control values in agricultural land (the Cd concentration threshold is 1.5 mg/kg, 2.0 mg/kg, 3.0 mg/kg, and 4.0 mg/kg in soil with pH ≤ 5.5, 5.5 < pH ≤ 6.5, 6.5 < pH ≤ 7.5, and pH > 7.5, respectively), the cultivation of edible agricultural products is banned, and remediation is essential. Therefore, addressing the issue of Cd-polluted soil is crucial to ensure food safety and promote the sustainable growth of agriculture. Developing techniques for remediating Cd pollution in a safe and efficient way has become a new research hotspot. Microbial remediation, which refers to the process in which microorganisms change the translocation characteristics and forms of heavy metals in the environment by fixing, moving, or transforming metals, can effectively remediate heavy metal-contaminated soil [12]. As a new technology, microbial remediation compensates for the limitations of traditional physical, chemical, and phytoremediation methods, such as high cost, long cycles, secondary pollution, and regional dependence. Thus, this low-cost, straightforward, and eco-friendly technology has a high application potential [13].

Macrofungi have been shown to have strong capacities for heavy metal adsorption and accumulation; for instance, 141.6–3500 mg/kg Cd was detected in the mycelia or fruiting bodies of *Cystoderma carcharias* [14], *Lentinula edodes* [15], and *Ganoderma lucidum* [16]. Furthermore, some studies have found that oyster mushrooms exhibit a high tolerance to Cd [17,18,19,20], indicating that *Pleurotus ostreatus* can be used as an effective biosorption material due to its ability to absorb 15.6 mg/kg Cd and 8.9 mg/kg Cr from soil [20]. The impacts of stress from heavy metals on microorganisms include alterations in the nucleic acid structure, damage to cell membranes, disturbance of physiological function, oxidative phosphorylation, and inhibition of enzyme activity, resulting in the denaturation of proteins, lipid peroxidation, and alteration of osmotic balance and ultimately affecting microorganism morphology, metabolism, and growth [13,21,22]. The ability of macrofungi to withstand heavy metals is attributed to the variety of detoxification mechanisms that these organisms have developed to keep homeostasis and combat the harm of heavy metals. First, the cell wall acts as the primary barrier for the penetration of heavy metals into cells. Specifically, the cell wall can release amino acids, organic acids, proteins, and other substances that can immobilize heavy metals to prevent them from damaging the intracellular structure [23]. Furthermore, the fungal cell wall is composed of chitin, lipids, inorganic ions, polyphosphates, nitrogenous polysaccharides, and proteins, which allow for active absorption, intracellular and extracellular precipitation, and the valence transformation of heavy metals, improving the tolerance of mycelia [24]. Second, heavy metal ions that are not immobilized by the cell wall bind to sugars, amino acids, proteins, etc., in the cell to reduce toxicity [25,26]. In a study on *Pleurotus platypus*, polysaccharides detected in the cell wall were shown to bind to Cd for detoxification [27]. Additionally, *L. edodes* employs amino acids such as Glu, Asp, and His to bind effectively to Cd [28]. Additionally, LECBP, a Cd-binding protein derived from *L. edodes*, has been used to adsorb Cd in *Escherichia coli* [29]. Furthermore, the antioxidant system has a crucial function in detoxification and can eliminate reactive oxygen species (ROS) induced by heavy metal stress, consequently enhancing the resistance of macrofungi [30]. Antioxidase (POD, SOD, CAT, etc.) and antioxidants (GSH, etc.) were found to be key factors involved in Cd detoxification in *P. ostreatus* and *Xerula radicata* [20,31]. Moreover, their rapid development of fruiting bodies and their high biomass make macrofungi ideal for use in microbial remediation.

According to our previous studies, *Stropharia rugosoannulata* Farlow ex Murrill showed the capacity for Cd accumulation [32]. After exposure to Cd stress at concentrations of 0.2 mg/L and 2 mg/L for 21 days, Cd concentrations in the mycelia were 0.19 mg/kg and 71.09 mg/kg, respectively. The Cd removal rates in the solution were 58.50% and 29.40%, respectively, indicating that *S. rugosoannulata* has the potential to be utilized as a substance for soil remediation. By performing an integrated analysis of physiological and proteomic data, we conducted an initial investigation into the mechanism of Cd tolerance and found that pathways related to amino acid metabolism, the antioxidant system, and energy metabolism responded in different ways under different levels of Cd stress. Nonetheless, the reaction to abiotic stress relies on diverse interactions, encompassing the interplay among genes, proteins, and metabolites [33]. Although we have some explanations for the Cd detoxification mechanisms based on analyses at the physiological and protein levels, whether these changes in DNA and proteins regulate downstream metabolites is unclear. Therefore, the application of metabolomics can provide an accurate understanding of the biological metabolic system [34]. Hence, the utilization of metabolomic analysis in conjunction with genomics and proteomics data can enhance and supplement the combined omics approach, facilitating a more holistic understanding of the interactions among diverse metabolic pathways. Omics studies, which can provide valuable information for obtaining a thorough understanding of the molecular mechanisms that contribute to the tolerance of plants to abiotic stress [35], have been applied in research on Cd tolerance mechanisms in some plants. The protein, metabolite, and ionomic responses involved in Cd hypertolerance in the leaf and root tissues of *Sedum alfredii* exposed to 50 µM Cd have been examined using isobaric tags for relative and absolute quantification (iTRAQ) marked proteomics, nontarget metabonomics, and ICP−MS technology, and the proteins involved in photosynthetic pathways and sulfur- and GSH-related metabolism were identified as key factors involved in Cd detoxification [36]. The effects of 25 μM and 100 μM Cd stresses on photosynthesis in *Brassica juncea* (Indian mustard) have been investigated through proteomic and metabolomic analyses, and several enzymes and metabolic pathways contributing to the observed alterations in photosynthesis have been identified [37]. Nevertheless, little omics information has been found for the responses of *S. rugosoannulata* to Cd stress.

In this study, the metabolite differences of *S. rugosoannulata* mycelia under different Cd stress levels were compared via LC−MS untargeted metabolomics analysis. Furthermore, the results were combined with those from our previous proteomics analysis to conduct an integrated proteomics and metabolomics analysis; therefore, the proteins, metabolites, and pathways related to the response of mycelia to Cd stress were screened. A comprehensive understanding of the mechanisms underlying the responses of *S. rugosoannulata* mycelia to Cd stress can provide a theoretical basis for its application in soil remediation.

## 2. Materials and Methods

### 2.1. Culture and Treatment of Mycelia

*S. rugosoannulata* strain Zhaoyang was acquired from the Strain Preservation Center of the Institute of Edible Fungi, Shanghai Academy of Agricultural Sciences in China. For metabolite analysis, mycelia were cultured and collected in liquid potato dextrose broth (PDB) supplemented with Cd^2+^ as CdCl_2_·2.5 H_2_O (AR) at concentrations of 0 mg/L, 0.2 mg/L (low), and 2.0 mg/L (high) (forming the control, Cd 0.2, and Cd 2 treatment groups). The conical flasks treated with PDB and Cd^2+^ were tightly sealed using sealing film and subsequently sterilized at a temperature of 121 °C for 20 min. The strain was cultured for 21 days in solid potato dextrose agar (PDA) without Cd and then crushed in PDB (100 mL of PDB per solid plate). Subsequently, 10 mL of the strain was crushed with PDB, inoculated in 100 mL of treated liquid medium, and cultured in a shaker for 21 days (25 °C, 150 g). Each treatment was performed in triplicate. After treatment, the cultured mycelia were filtered from liquid culture. To eliminate Cd that had adsorbed onto the surface of the mycelia, the mycelia were submerged in a solution of 20 mM EDTA-Na_2_ for 15 min. Subsequently, the sections were rinsed and dried. The collected mycelia were rapidly frozen in liquid nitrogen and preserved at a temperature of −80 °C for future use.

### 2.2. Sample Preparation for LC−MS Untargeted Metabolomic Analysis

The weighed sample (20 mg) was transferred to an Eppendorf tube (1.5 mL) and ground with a pair of steel balls. Twenty milliliters of 2-chloro-L-phenylalanine (0.3 mg/mL) was added to methanol as an internal standard. Then, 1 mL of the mixed solution (7 methanol/3 water, *v/v*) was added to each sample, and the mixture was incubated for 2 min at −20 °C. The specimens were pulverized for 2 min at a frequency of 60 Hz, vortexed, ultrasonicated at room temperature for 30 min, and subsequently maintained at −20 °C for 20 min. After centrifugation at a speed of 13,000 revolutions per minute for 10 min (4 °C), 300 μL of the supernatant was obtained and freeze-dried. Then, 400 μL of mixed solution (1 methanol/4 water, *v/v*) was added, and the mixture was vortexed for 30 s, following ultrasonication at ambient temperature for 2 min. The mixture was centrifuged again (under the same conditions as above). Next, an injection syringe was used to collect 150 μL of supernatant from each tube. Microfilters (0.22 μm) were used for filtration, and the samples were transferred to LC vials and maintained at −80 °C for further LC–MS analysis. The quality control (QC) samples were pooled into aliquots of each sample.

### 2.3. LC−MS Analysis

The metabolic profiles of the mycelia were analyzed using an ACQUITY UHPLC system (Waters Corporation, Milford, MA, USA) combined with an AB SCIEX Triple TOF 5600 System (AB SCIEX, Framingham, MA, USA). An ACQUITY UPLC BEH C18 column (1.7 μm, 2.1 × 100 mm) was used. The elution system and process for gradient separation were as described previously by Wang et al. [38]. Throughout the analysis, the samples were maintained at a temperature of 4 °C. The gathering of data was carried out using a combination of a full scan mode (with m/z ranging from 125 to 1000) and an IDA mode. To evaluate repeatability, QCs were injected once every 10 samples during the analysis.

### 2.4. Data Preprocessing and Analysis

Progenesis QI and data processing software (http://www.hmdb.ca/) (Waters Corporation, Milford, MA, USA) were used for the raw LC–MS data analysis and metabolite identification. The combined data were imported to the R package. To visualize the metabolic changes among the treatments, principal component analysis (PCA) and (orthogonal) partial least-squares-discriminant analysis (O) PLS-DA were carried out after mean centering and Pareto variance scaling, respectively. Hotelling’s T2 region (ellipse in the models) was used to define the 95% confidence interval of the modeled variation. The selection of differentially expressed metabolites (DEMs) depended on the combination of a statistically significant threshold of variable importance in the projection (VIP values) from the OPLS-DA model and *p* values from a two-tailed Student’s t test on the normalized peak areas. The screening conditions were VIP > 1.0 and *p* < 0.05. Furthermore, the Kyoto Encyclopedia of Genes and Genomes (KEGG) database was utilized to search for metabolite pathways.

### 2.5. Statistical Analysis

SAS software (version 9.1; SAS Institute, Inc., Carly, NC, USA) was used to perform the statistical analyses. To identify the differences among treatment groups, the least significant difference (LSD) test was used based on a significance level of 0.05.

## 3. Results

### 3.1. Cd Induced Metabolic Response in S. rugosoannulata Mycelia

**Figure 1:**
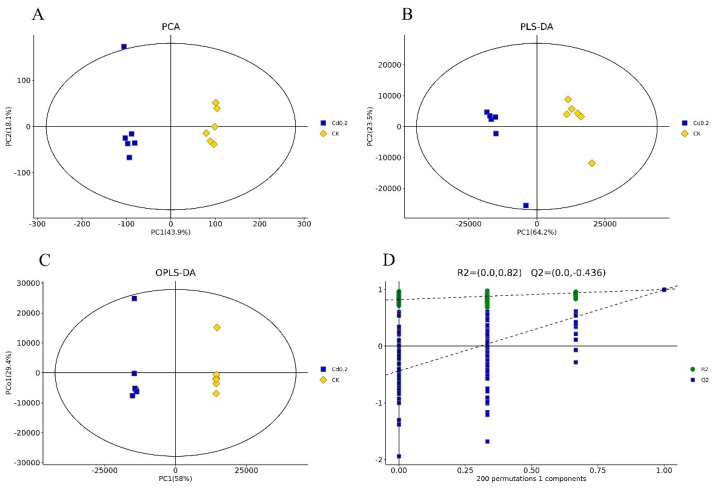
Multivariate statistical score and response-sorting test diagrams for the 0.2 mg/L Cd-treated (Cd 0.2) and control (CK) mycelia. (**A**): PCA; (**B**): PLS-DA; (**C**): OPLS-DA; (**D**): response-sorting test of the OPLS-DA model.

**Figure 2:**
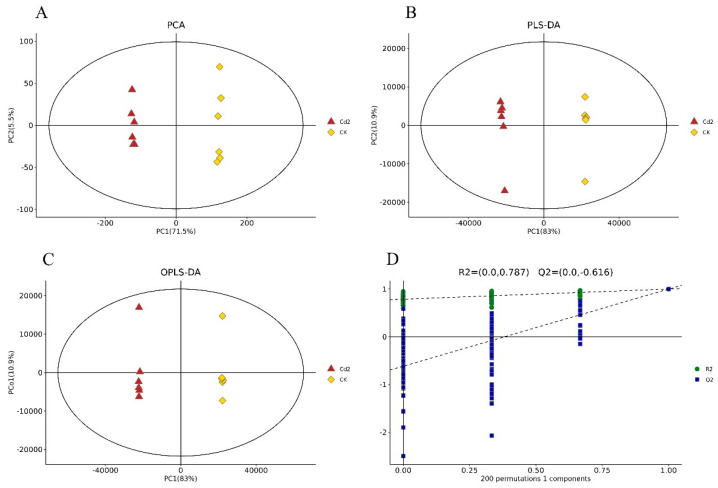
Multivariate statistical score and response-sorting test diagrams for the 2 mg/L Cd-treated (Cd 2) and control (CK) mycelia. (**A**): PCA; (**B**): PLS-DA; (**C**): OPLS-DA; (**D**): response-sorting test of the OPLS-DA model.

A PCA of the individual replicates of the groups in each comparison was conducted, and the two principal components explained 62.0% and 77.0% of the variation between the Cd 0.2 treatment group and the control group (Figure 1A) and between the Cd 2 treatment and the control group (Figure 2A), respectively. Through PLS-DA, the two principal components explained 87.7% and 93.9% of the variation between the two compared groups (Figure 1B and Figure 2B), respectively. The OPLS-DA results showed that the two principal components explained 87.4% and 93.9% of the variation between the two compared groups (Figure 1C and Figure 2C), respectively. In all three models, noticeable separation was observed between the two compared groups. The quality of the model was assessed using sevenfold validation and 200 permutation tests for response (RPTs). The validity of the model after cross-validation is shown in Figure 1D and Figure 2D. These results indicated that Cd stress altered the metabolite profiles of *S. rugosoannulata* mycelia.

### 3.2. Identification of DEMs

**Figure 3:**
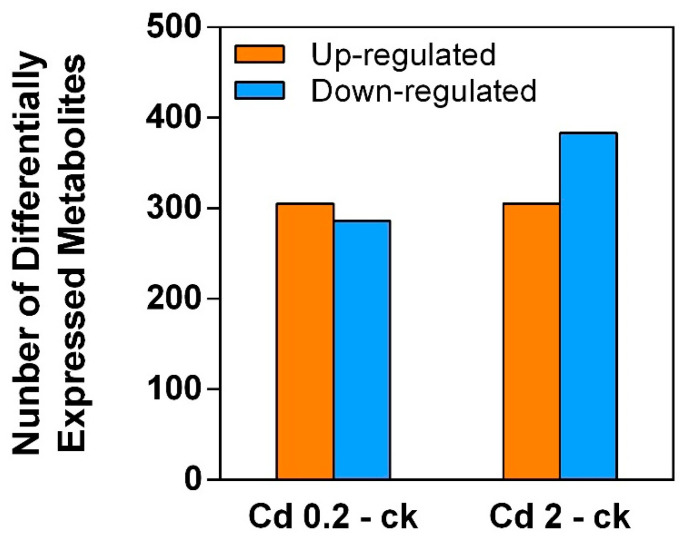
DEMs (significantly up- and downregulated) in *S. rugosoannulata* mycelia exposed to Cd stress identified via LC−MS untargeted metabolomics analysis. Cd 0.2-ck and Cd 2-ck represent the up/downregulated DEMs identified from the 0.2 mg/L Cd treatment group vs. control group comparison and the Cd 2-ck treatment vs. control group comparison, respectively.

**Figure 4:**
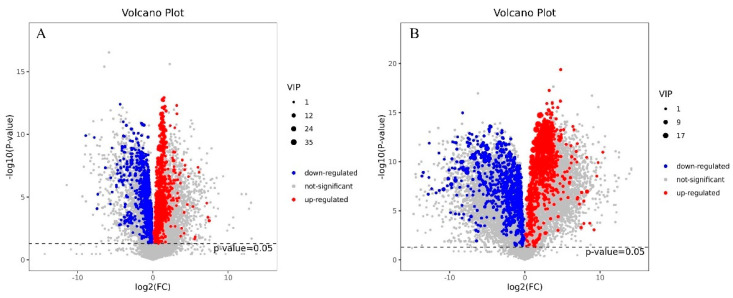
Volcano plot of DEMs identified in *S. rugosoannulata* mycelia in response to Cd. (**A**): DEMs identified from the comparison of 0.2 mg/L Cd-treated mycelia with control mycelia; (**B**): DEMs identified from the comparison of 2 mg/L Cd-treated mycelia with control mycelia. The log2(FC) is represented on the *x*-axis, and the −log10 (*p* value) is represented on the *y*-axis. The VIP value increases as the dot area decreases, indicating increases in the significance for the difference.

The metabolite changes were identified by LC–MS. As shown in Figure 3 and Figure 4, a total of 591 DEMs (305 upregulated and 286 downregulated) were identified in the mycelia by comparing the 0.2 mg/L Cd treatment and control (ck) groups. A total of 688 DEMs (305 upregulated and 383 downregulated) were identified between the 2 mg/L Cd treatment and control (ck) groups. Tables S1 and S2 display detailed information of the DEMs identified from the two comparisons. The results implied that the quantity of DEMs increased after Cd treatment in a concentration-dependent manner.

**Figure 5:**
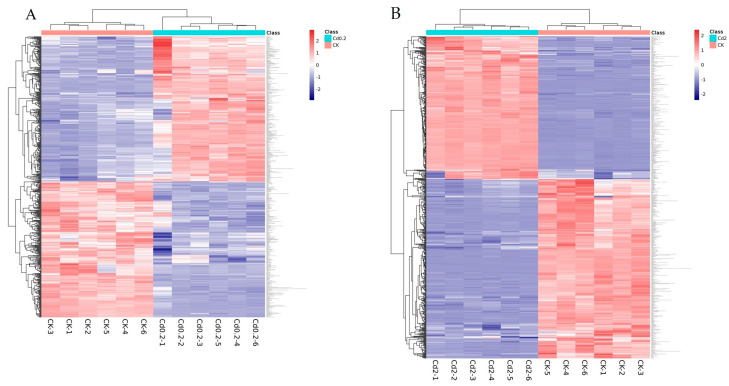
Hierarchical cluster analysis of the changed metabolite pools. Hierarchical trees were created based on the changes in metabolites in *S. rugosoannulata* mycelia exposed to Cd. (**A**): DEMs identified from the comparison of 0.2 mg/L Cd-treated mycelia with control mycelia; (**B**): DEMs identified from the comparison of 2 mg/L Cd-treated mycelia with control mycelia. The different treatments are represented by columns, and the rows indicate different metabolites. Red and blue indicate metabolites found at increased and decreased levels, respectively.

The metabolome profiles of the mycelia under low- and high-Cd stress were analyzed via hierarchical cluster analysis (Figure 5). The results indicated that the control, 0.2 mg/L Cd, and 2 mg/L Cd groups were segregated based on their metabolite responses, suggesting that the mycelia responded differently to low and high levels of Cd at the metabolite level.

**Figure 6:**
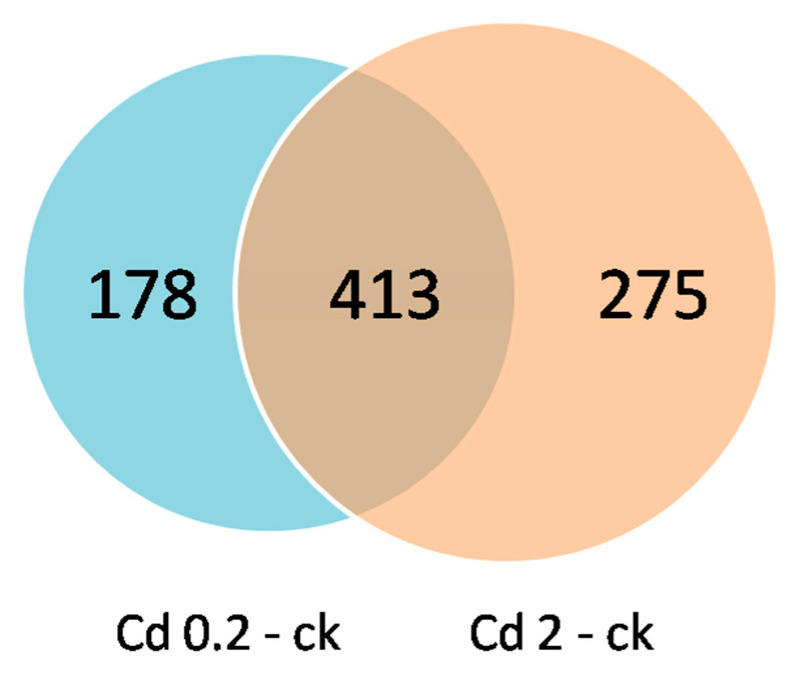
Venn diagram analysis of DEMs in *S. rugosoannulata* mycelia exposed to Cd identified via LC−MS untargeted metabolomics analysis. Cd 0.2-ck and Cd 2-ck represent the up/downregulated DEMs identified from the 0.2 mg/L Cd treatment group vs. the control group comparison and the Cd 2-ck treatment vs. the control group comparison, respectively.

A Venn diagram analysis revealed that 413 DEMs were identified in both the low-Cd vs. control group and high-Cd vs. control group comparisons, implying that these metabolites may play vital roles in the Cd detoxification process in mycelia (Figure 6).

### 3.3. Kyoto Encyclopedia of Genes and Genomes (KEGG) Metabolic Pathway Enrichment Analysis of the DEMs

**Figure 7:**
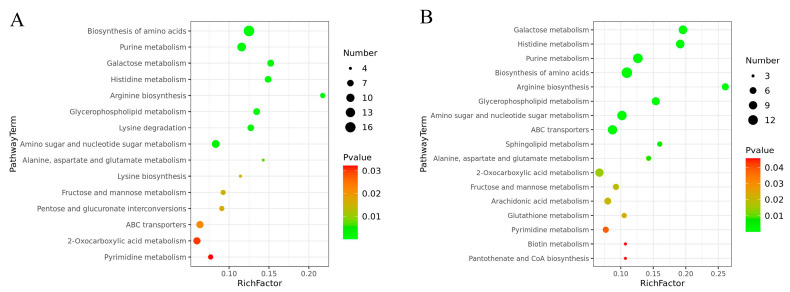
KEGG pathway enrichment of DEMs in *S. rugosoannulata* mycelia exposed to Cd. (**A**): DEMs identified from the comparison of the 0.2 mg/L Cd treatment group with the control group; (**B**): DEMs identified from the comparison of the 2 mg/L Cd treatment group with the control group. The KEGG pathways are symbolized by the circles, the pathway name is shown on the *y*-axis, and the enrichment score is shown on the *x*-axis. The enrichment score reflects the proportion of DEMs annotated to the pathway among all metabolites annotated into the pathway. The color of the circle represents the *p* value. A lower *p* value indicates a greater reliability of the enrichment of DEMs within a given pathway. The number of DEMs enriched in the pathway is represented by the size of the circle, and a larger circle indicates a higher number of metabolites.

All of the DEMs that responded to the two levels of Cd stress were mapped to the KEGG database for an analysis of the associated pathways. This analysis revealed that 64 and 62 pathways were mapped with DEMs identified from the comparison of the 0.2 mg/L and 2 mg/L Cd treatment groups, respectively, with the control group (Tables S3 and S4). The number of significantly enriched pathways (*p* < 0.05) increased from 15 to 17 with increases in the Cd concentration (Figure 7). The KEGG pathways associated with the DEMs identified from both the two comparisons included the biosynthesis of amino acids, purine metabolism, galactose metabolism, histidine metabolism, arginine biosynthesis, glycerophospholipid metabolism, alanine, aspartate and glutamate metabolism, amino sugar and nucleotide sugar metabolism, fructose and mannose metabolism, 2-oxocarboxylic acid metabolism, and ABC transporters and pyrimidine metabolism, indicating that these are the main metabolic pathways involved in the physiological processes that occur in mycelia under Cd stress. However, lysine degradation, lysine biosynthesis, and pentose and glucuronate interconversions were significantly enriched only under 0.2 mg/L Cd stress, whereas arachidonic acid metabolism, sphingolipid metabolism, glutathione (GSH) metabolism, biotin metabolism, and pantothenate and CoA biosynthesis were significantly enriched under 2 mg/L Cd stress, indicating that mycelia utilize different metabolic response mechanisms to different levels of Cd stress and that the alterations in these pathways may be related to the dose of Cd stress.

### 3.4. Integrated Proteomic and Metabolomic Analyses of S. rugosoannulata Mycelia in Response to Cd Stress

In the previous investigation, we analyzed the proteomic response of *S. rugosoannulata* mycelia under the same stress treatments, and we combined these data with the changes in the metabolome identified in this study to conduct KEGG mapping-based integrated proteomic and metabolomic analyses. The related DEPs, DEMs, and metabolic pathways are shown in Table 1, Tables S5 and S6. Low-Cd stress induced DEPs and DEMs associated with six metabolic pathways, and only one of these pathways (glycerophospholipid metabolism) was significantly enriched (*p* < 0.05) according to both proteomic and metabolomic analyses. Forty-two common pathways were identified under high-Cd treatment, whereas six pathways, including arginine biosynthesis, galactose metabolism, alanine, aspartate and glutamate metabolism, histidine metabolism, glutathione metabolism, and arachidonic acid metabolism, were significantly enriched (*p* < 0.05) according to the integrated proteomic and metabolomic analysis. These findings indicate that these six pathways play important roles in helping *S. rugosoannulata* mycelia cope with high levels of Cd stress.

**Figure 8:**
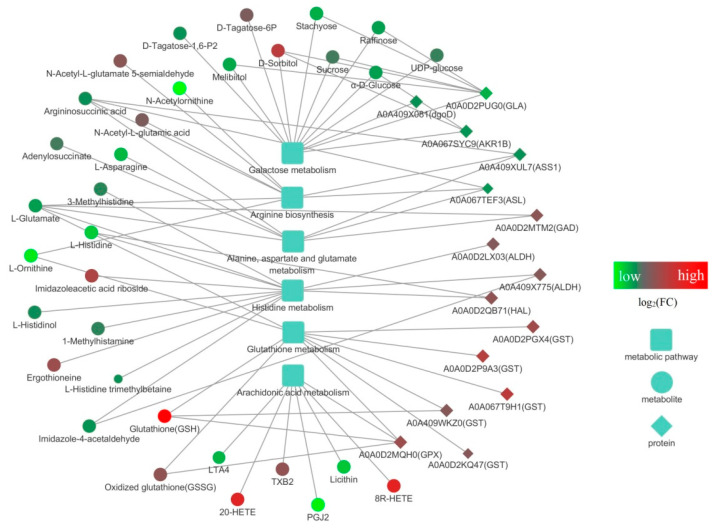
Network analysis of the DEPs, DEMs, and pathways associated with *S. rugosoannulata* mycelia. The analysis was performed with Cytoscape software (V 3.7.2). The squares represent metabolic pathways, the circles represent DEMs, the rhombuses represent DEPs, and the solid lines represent interactions. The size of the shape represents the *p* value; a larger shape indicates a smaller *p* value. The color of the shape represents log2(FC), and the change in color from green to red indicates a low to high log2(FC).

The six significantly enriched common pathways identified under high-Cd stress were further analyzed. An interaction diagram was constructed by integrating the DEPs, DEMs, and pathways (Figure 8). The network includes six metabolic pathways and their related 33 DEMs and 15 DEPs. Among the DEMs, GSH, 8R-HETE, 20-HETE, and D-sorbitol, which are related to glutamate metabolism, arachidonic acid metabolism, and galactose metabolism, had the highest fold changes in expression, whereas L-asparagine, lecithin, L-histidine, L-ornithine, PGJ2, and N-acetylornithine, which are related to five pathways (excluding galactose metabolism), exhibited the lowest fold changes in expression. Among the DEPs, A0A067T9H1 (GST), A0A0D2P9A3 (GST), A0A0D2PGX4 (GST), and A0A0D2MQH0 (GPX), which are involved in glutamate metabolism, showed the highest fold changes in expression, whereas A0A067SYC9 (AKR1B), A0A067TEF3 (ASL), and A0A0D2PUG0 (GLA), which are related to galactose metabolism, arginine biosynthesis, alanine, aspartate, and glutamate metabolism, exhibited the highest fold changes in expression. Furthermore, a total of 13 DEMs and DEPs participated in the galactose metabolism pathway, surpassing the number observed in other pathways. Notably, L-glutamate was involved in four pathways and simultaneously interacted with the GAD protein. In addition to directly interacting with the GPX and GST proteins, GSH exhibited the highest fold change in expression (8.28). The GLA protein directly reacted with D-sorbitol, melibiitol, raffinose, stachyose, and sucrose, all of which regulate galactose metabolism in *S. rugosoannulata* mycelia. The interactions between GPX protein and glutamate metabolism, arachidonic acid metabolism, GSH, and GSSG revealed that glutamate regulation might be the main mechanism by which *S. rugosoannulata* mycelia prevent Cd toxicity.

### 3.5. Pathway Analysis of the DEMs and DEPs

**Figure 9:**
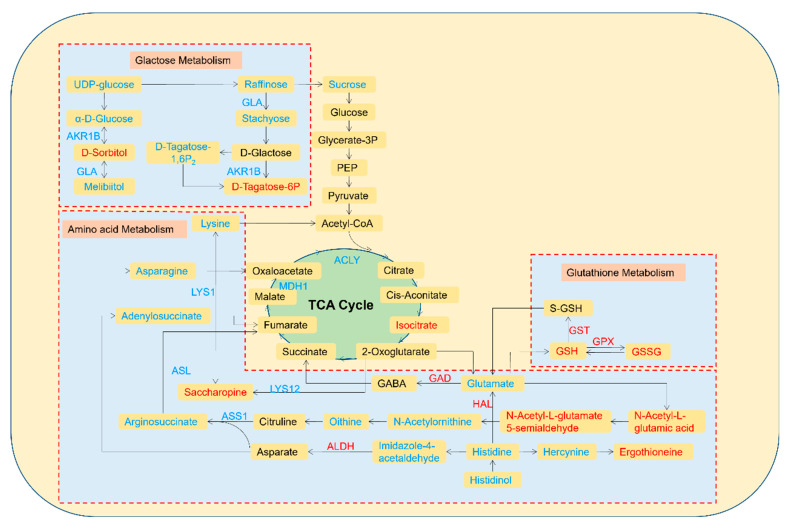
Main biological pathways involved in the response of *S. rugosoannulata* mycelia to high Cd stress. The yellow boxes represent metabolites, the arrows indicate DEPs, and the red and blue font indicate up- and downregulation, respectively.

To investigate the molecular mechanisms by which *S. rugosoannulata* mycelia regulate and adapt to high levels of Cd stress (2 mg/L), a detailed metabolic pathway diagram was created using the KEGG pathways, incorporating the important DEPs and metabolites. As depicted in Figure 9, the diagram was mapped with 13 DEPs and 28 DEMs. Under high-Cd stress, the DEPs and DEMs in the mycelia were mainly associated with galactose metabolism, some amino acid metabolism, and GSH metabolism.

Most of the DEPs and DEMs in the galactose metabolism pathway were downregulated. The downregulation of the GLA and AKR1B proteins led to reductions in raffinose, stachyose, D-tagatose-1,6-P2, α-d-glucose, and melibiitol. Due to the interactions between proteins and metabolites, the levels of D-sorbitol and D-tagatose-6P increased. Moreover, the levels of UDP-glucose and sucrose, the key metabolites of galactose metabolism, decreased, which also resulted in the subsequent reductions in EMP and the TCA cycle.

The effects of high-Cd stress on amino acids involved mainly lysine, asparagine, histidine, ornithine, and glutamate, as well as intermediates and proteins in the related metabolic pathways. All abovementioned amino acids were downregulated; among the intermediate products, significant downregulation of adenylosuccinate, argininosuccinate, N-acetylornithine, imidazole-4-acetaldehyde, histidinol and hercynine (which are involved in alanine), aspartate and glutamate metabolism, arginine biosynthesis, and histidine metabolism, was detected. Other intermediate products, including saccharopine, N-acetyl-l-glutamate 5-semialdehyde, N-acetyl-l-glutamic acid and ergothioneine (which are involved in lysine synthesis and metabolism), arginine biosynthesis, and histidine metabolism, were upregulated. In addition, the expression of LYS1, ASL, LYS12, and ASS1 was downregulated, whereas the expression of GAD, HAL, and ALDH was upregulated, and these alterations directly or indirectly regulated the changes in amino acids and intermediate products in the pathways.

Under a high Cd stress, GSH metabolism, which is an important part of the antioxidant system, was upregulated. GSH was first upregulated, and FC reached 8.28, which was the highest among those of all the DEMs. The upregulation of GPX expression promoted the transformation of GSH to GSSG, resulting in a significant increase in the GSSG level. Five GST proteins were upregulated simultaneously, which promoted the transformation between GSH, S-GSH, and glutamate. These results indicate that a high level of Cd stress stimulates the activation of the antioxidant system in mycelia and that GSH metabolism plays a crucial role in this mechanism.

## 4. Discussion

As a nonessential heavy metal to organisms, Cd is extremely toxic. Cd can affect the metabolic system, inhibit growth and development, and lead to a decrease in biomass. In recent years, integrated multiomics analysis has been utilized to investigate plant resistance to various abiotic stresses [39]. However, few studies have investigated the tolerance mechanisms of *S. rugosoannulata* to Cd stress. In this research, the molecular mechanism of *S. rugosoannulata* mycelia exposed to different levels of Cd was investigated through integrated proteomics and metabolomics analyses.

### 4.1. Differential Response Mechanisms Are Activated in Mycelia under Different Levels of Cd Stress

The high biotoxicity of Cd can result in permanent harm to tissues, which, in turn, triggers stress responses in the organism. In this study, the metabolomes of *S. rugosoannulata* mycelia exposed to low (0.2 mg/L) and high (2 mg/L) Cd concentrations were compared with those of control mycelia, and combined with the results from our previous proteomic analysis under the same treatment conditions, we performed an integrated analysis. According to the identified DEPs, DEMs and their related, significantly enriched metabolic pathways, as well as low and high concentrations of Cd, induced different stress response mechanisms in mycelia. This result is consistent with the findings from our previous studies on the response of the antioxidant system and proteome, which showed that mycelia were insensitive to low-Cd stress and maintained normal growth, whereas high-Cd stress inhibited growth, and strong Cd resistance led to high Cd accumulation in mycelia [32]. The mycelia of *Fusarium* pathogens exhibited similar phenomena when exposed to Cd (10–75 ppm) [40]. Some plants exhibit a “low promotion and high inhibition” relationship in the presence of different concentrations of heavy metal stress: a low heavy metal concentration promotes plant growth and increases the biomass, and a high concentration inhibits growth and decreases the biomass [41]. Under various abiotic stresses, most plants do not have specialized resistant organs but rather exhibit enhanced morphological or physiological characteristics to cope with changes in the environment. The phenomenon in which such organisms adjust their morphological characteristics before pollution enters them to adapt to the stressed environment is called preadaptation. Changes in plant morphology, including biomass, under stress conditions are part of preadaptation, which is an important biological adaptation to pollution [42]. Studies on fungi (*Zygosaccharomyces rouxii*) [43] and bacteria (*Salmonella enterica*) [44] have also shown that preadaptation helps to improve their resistance to adverse conditions such as salt stress and the bile environment. In our study, the finding that mycelia do not exhibit sensitivity to low-Cd stress may also be a manifestation of preadaptation.

### 4.2. Response Mechanism of Mycelia under Low-Cd Stress Based on Integrated Proteomic and Metabolomic Analyses

Integrated proteomic and metabolomic analyses of the mycelia were also conducted, and only the glycerophospholipid metabolism pathway was significantly enriched in the mycelia exposed to a low Cd concentration, suggesting that the mycelia were insensitive to low-Cd stress. Research conducted on chickpea plants and humans has shown that exposure to Cd also results in disorders of glycerophospholipid metabolism, indicating that when organisms are subjected to heavy-metal stress, they can adapt to adverse environmental effects by regulating lipid changes in tissues [45,46]. Glycerophospholipids are the primary components of cell membranes and constitute the basic scaffold of cell membranes. They can maintain the integrity and stability of cell membranes, protect biomolecules inside cells from disturbance by the external environment, and regulate the entry and exit of substances to maintain the balance of the internal and external environments. Glycerophospholipids also have regulatory functions in response to environmental changes, energy conversion and signal transduction, cell apoptosis, oxidative stress defense, and other biological processes [47,48]. These findings align with our previous research. According to the results from the analyses of the Cd accumulation capacity, growth, and cell membrane oxidative damage in mycelia, MDA content was induced under low-Cd stress because even a low concentration of Cd can disturb glycerophospholipid metabolism in mycelia; thus, the structure of the cell membrane was affected, and a certain degree of oxidative damage ultimately occurred [32]. However, at this time, the H_2_O_2_ level decreased even though the antioxidant system was not completely activated, suggesting that a low Cd stress had little effect on the physiological process of mycelia. On the other hand, the analysis of the metabolome revealed that the disturbed metabolic pathway of the mycelia when exposed to low-Cd stress primarily included amino acid and glucose metabolisms, combined with mycelial growth and physiological indicators in our previous research [32]; it is indicated that the mycelia under a low Cd stress adopted an active defense strategy, which was consistent with research on the response of cucumber to perfluorooctanoic acid [49]. Mycelia ultimately avoid cellular damage through such a strategy, which also accounts for the low Cd accumulation and normal growth of mycelia.

### 4.3. Response Mechanism of Mycelia under High-Cd Stress Based on Integrated Proteomic and Metabolomic Analyses

Six pathways were significantly enriched under a high-Cd stress, as revealed by integrated proteomic and metabolomic analyses. According to the comprehensive metabolic pathway diagram, 13 DEPs and 28 DEMs, which are involved in amino acid, glucose, and GSH metabolism, contributed to the tolerance of mycelia to high-Cd stress. A high concentration of Cd induced changes in eight DEMs and two DEPs involved in the galactose metabolism pathway, suggesting changes in carbon fixation in the mycelia. The galactose metabolic pathway represents a process of glycometabolism that involves sucrose, trehalose, galactose, raffinose, etc. [50]. Previous studies have shown that glycometabolism is the key factor in resisting abiotic stress and providing an energy supply for normal plant growth [51]. For instance, under water shortage conditions, sucrose can be translocated to the tips of plant roots to enhance root development [52], and oligosaccharides in different species exhibit similar response patterns under cold stress [53] and salt stress [54]. Carbohydrate accumulation in plant tissue primarily serves to maintain the osmotic equilibrium of cells [55]. Nevertheless, plants exposed to specific metal-based pollutants typically do not encounter such disruptions [56]. In this study, down the regulation of GLA and AKR1B proteins resulted in decreases in UDP-glucose, raffinose, stachyose, α-d-glucose, melibiitol, and D-tagatose-1,6P2 and increases in D-sorbitol and D-tagatose-6P, ultimately reducing the amount of sucrose entering the glycolysis pathway. Therefore, under high-Cd stress, mycelia may reduce the accumulation of carbohydrates to improve tolerance through the regulation of key proteins and metabolites in the galactose pathway, which may also be the main reason for the inhibition of their growth.

In addition, a high-Cd stress disrupted three amino acid metabolic pathways. The involvement of these pathways, which are central to nitrogen and carbohydrate metabolisms, may also explain how a high-Cd stress inhibits mycelial growth and nutrient translocation. Figure 9 shows that due to the regulation of enzymes such as LYS1, ASL, ASS1, HAL, and ALDH, the expression of several native amino acids, including lysine, asparagine, ornithine, histidine, and glutamate, and their intermediates decreased, indicating that Cd stress interfered with the primary metabolism of the mycelia. These amino acids are vital in metabolic networks and tolerance to heavy metals. For instance, aspartic acid and glutamate are essential for the biosynthesis of many other amino acids; several amino acids, including lysine, can bind to Cd^2+^, reducing its toxicity to organisms; and polylysine, composed of lysine residues, has been proven to be useful as an antidote to heavy metals [57,58]. In contrast, glutamic acid, ornithine, and proline participate in the main network of nitrogen metabolism. During this process, intermediate products, such as nitric oxide (NO) and gamma-aminobutyric acid (GABA), can be produced and have been proven to support the growth, development, and abiotic stress resistance of plants [59,60,61,62,63]. The downregulation of glutamate may be due to the upregulation of GAD, which accelerates its conversion into GABA. According to studies on rice [64], barley [65], and Compositae plants [66], the levels of amino acids increase in the presence of Cd, which differs from the findings of the present study. This difference could be attributed to the impairment in galactose metabolism induced by a high-Cd stress, which resulted in insufficient energy for amino acid metabolism. Consequently, amino acid metabolism was directly weakened. Because native amino acids are components of proteins, their reduction may inhibit protein biosynthesis and, thus, plant growth, whereas reductions in carbohydrates and amino acids also compromise the nutritional value of the organism [56].

According to our results, another noteworthy pathway that functions under a high-Cd stress is the GSH metabolic pathway, which was significantly enriched only under high-Cd stress conditions. Increases in the Cd concentration completely activated the antioxidant system, and GSH was the metabolite with the highest fold change (8.28). This finding is consistent with the findings of our previous research, which showed that the GSH levels notably increased under a high-Cd stress and that the H_2_O_2_ levels continued to decrease in comparison to those under a low-Cd stress. Ultimately, the mycelia successfully sustained growth and accumulated 71.09 mg/kg Cd in the presence of a 2 mg/L Cd stress. The involvement of the antioxidant system is attributed to these results. As shown in Figure 9, the levels of GSH and GSSG increased, and the upregulation of GST and GPX contributed to the maintenance of the dynamic balance among S-GSH, GSH, and GSSG. Moreover, the decrease in the glutamate concentration was also due to the conversion of high amounts of glutamate to GSH. As a nonprotein mercaptan, GSH is a crucial antioxidant involved in redox homeostasis [67,68,69]. Many studies have demonstrated that fungi and plants mitigate oxidative stress by generating an abundance of antioxidants, such as GSH, ASA, and polyphenols [70,71,72]. GSH can eliminate heavy metals in tissues through two methods: first, GSH can directly capture ions on the sulfur group of the enzyme protein and bind to it; second, as a precursor, an increase in GSH facilitates the synthesis of phytochelatin, which can chelate heavy metals and be translocated to the vacuole for isolation or exclusion outside the cell by transporters [73,74]. The results of multiomics and physiological analyses demonstrated that GSH and ASA were crucial for H_2_O_2_ clearance, which is consistent with the results found in *Brassica chinensis* L. [75,76]. When exposed to Cd stress, mycelia can increase the activity of antioxidant enzymes and upregulate the key proteins involved in the ASA-GSH cycle. These effects led to an increase in the GSH and ASA levels, which helped alleviate oxidative stress and the growth inhibition caused by Cd, ultimately reducing its toxicity. In summary, due to the common regulation of galactose metabolism, amino acid metabolism, and GSH metabolism pathways, the mycelia of *S. rugosoannulata* exhibit a certain tolerance to high concentrations of Cd. These results support the feasibility of soil remediation technology based on the Cd tolerance of *S. rugosoannulata* mycelia and establish a theoretical foundation for its application in the remediation of Cd-contaminated soil.

## 5. Conclusions

In this study, we conducted integrated proteomic and metabolomic analyses based on TMT and LC−MS techniques to explore the response mechanisms of the mycelia of *S. rugosoannulata* under 0.2 mg/L (low) and 2 mg/L (high) Cd stresses. Mycelia utilize different molecular mechanisms in response to different levels of Cd stress. According to the integrated analyses, only one pathway was significantly enriched by 0.2 mg/L Cd treatment, suggesting that the organism was not sensitive to a low-Cd stress. The Cd-induced cell membrane oxidative damage was attributed to the disturbance of glycerophospholipid metabolism, and mycelia under a low-Cd stress adopted an active defense strategy to avoid damage to their physiological function. Six pathways were found to be significantly enriched under a 2 mg/L Cd stress. The strong tolerance of mycelia to high-Cd stress is dependent mainly on the comprehensive regulation of galactose metabolism, amino acid metabolism, and the GSH metabolism pathways, which leads to the balance of nitrogen and carbohydrate metabolisms and to the alleviation of the oxidative stress and growth inhibition caused by Cd. These results support the feasibility of soil remediation technology based on the Cd tolerance of *S. rugosoannulata* mycelia and establish a theoretical foundation for its application in the remediation of Cd-contaminated soil.

## Figures and Tables

**Table 1:** Common pathways involved in integrated proteomic and metabolomic analyses *of S. rugosoannulata* mycelia under Cd stress.

Pathway Name	Proteomics		Metabolomics	
	Pathway ID	*p* Value	Pathway ID	*p* Value
Treatment with 0.2 mg/L Cd				
Glycerophospholipid metabolism	ko00564	0.0094522	sce00564	0.00048
Treatment with 2 mg/L Cd				
Galactose metabolism	ko00052	0.016538	sce00052	4.73 × 10^−6^
Arginine biosynthesis	ko00220	0.039474	sce00220	3.5 × 10^−5^
Alanine, aspartate, and glutamate metabolism	ko00250	0.034689	sce00250	0.007973
Histidine metabolism	ko00340	0.023705	sce00340	5.71 × 10^−6^
Glutathione metabolism	ko00480	0.00018	sce00480	0.023039
Arachidonic acid metabolism	ko00590	0.033405	sce00590	0.020527

## Data Availability

Data are contained within the article and Supplementary Materials.

## Supplementary Materials

The following supporting information can be downloaded at: https://www.mdpi.com/article/10.3390/jof10020134/s1, Table S1: The differential expression metabolites (DEMs) between 0.2 mg/L Cd treatment group and control group in *S. rugosoannulata* mycelia; Table S2: The differential expression metabolites (DEMs) between 2 mg/L Cd treatment group and control group in *S. rugosoannulata* mycelia; Table S3: Statistics of pathway enrichment in the KEGG pathway of DEMs in *S. rugosoannulata* mycelia under 0.2 mg/L Cd stress; Table S4: Statistics of pathway enrichment in the KEGG pathway of DEMs in *S. rugosoannulata* mycelia under 2 mg/L Cd stress; Table S5: Proteins and metabolites involved in common pathways of *S. rugosoannulata* mycelia under 0.2 mg/L Cd stress; Table S6: Proteins and metabolites involved in common pathways of *S. rugosoannulata* mycelia under 2 mg/L Cd stress.
